# Intra-osseous infiltration of adipose mesenchymal stromal cells and plasma rich in growth factors to treat acute full depth cartilage defects in a rabbit model: Serum osteoarthritis biomarkers and macroscopical assessment

**DOI:** 10.3389/fvets.2022.1057079

**Published:** 2022-12-20

**Authors:** Marta Torres-Torrillas, Elena Damiá, Pau Peláez, Laura Miguel-Pastor, Belén Cuervo, José J. Cerón, José M. Carrillo, Mónica Rubio, Joaquín J. Sopena

**Affiliations:** ^1^Bioregenerative Medicine and Applied Surgery Research Group, Department of Animal Medicine and Surgery, CEU Cardenal Herrera University, CEU Universities, Valencia, Spain; ^2^García Cugat Foundation, CEU-UCH Chair of Medicine and Regenerative Surgery, CEU Cardenal Herrera University, CEU Universities, Valencia, Spain; ^3^Interdisciplinary Laboratory of Clinical Analysis, Interlab-UMU, Regional Campus of International Excellence, Campus Mare Nostrum, University of Murcia, Murcia, Spain

**Keywords:** adipose mesenchymal stromal cells, platelet rich plasma, osteoarthritis, chondral defect, articular cartilage, biomarkers, bioregenerative therapies

## Abstract

**Introduction:**

Intra-articular infiltration of plasma rich in growth factors (PRGF) and adipose mesenchymal stromal cells (AMSCs) are known to inhibit osteoarthritis progression. However, in severely affected patients, the treatment cannot reach the deeper layers of the articular cartilage; thus, its potential is limited. To overcome this limitation, intra-osseous infiltrations have been suggested. The purpose of this study is to assess the impact of intra-osseous infiltration therapies on serum biomarkers of osteoarthritis and to assess cartilage regeneration macroscopically.

**Materials and methods:**

A total of 80 rabbits were divided into four groups based on the intra-osseous treatment administered on the day of surgery: control, PRGF, AMSCs and a combination of PRGF + AMSCs. In addition, all groups received a single intra-articular administration of PRGF on the same day. Serum biomarker levels were measured before infiltration and 28-, 56-, and 84-days post infiltration, and macroscopical assessment was conducted at 56- and 84-days follow-up post infiltration.

**Results:**

In the PRGF + AMSCs group, significantly lower concentrations of hyaluronic acid and type II collagen cleavage neoepitope were recorded at all time points during the study, followed by PRGF, AMSCs and control groups. Regarding macroscopical assessment, lower scores were obtained in PRGF + AMSCs group at all study times.

**Discussion:**

The results suggest that the combination of intra-articular PRGF with intra-osseous PRGF or AMSCs achieves better results in rabbits with acute chondral defects and that intra-osseous infiltration is a safe procedure.

## Introduction

Articular cartilage (AC) has a limited self-repair potential due to its avascularity and low cellular mitotic activity of chondrocytes. Although small chondral lesions may heal spontaneously, larger defects can lead to progressive joint degeneration and osteoarthritis (OA) (1). OA is the most commonly diagnosed joint disease in companion animals and affects over 20% of the canine population (2, 3). Moreover, although it can be diagnosed in cats as young as 1 year old, evidence of radiographic OA is greater in older cats, with 90% of cats over 12 years of age being affected (4, 5).

OA is characterized by degeneration of AC and progressive destruction of intra-articular (IA) structures, including subchondral bone (SB) and synovial membrane (SM) (6, 7). All these tissues play a vital role in joint homeostasis, and any disturbance of its balance results in AC and SB degeneration, osteophyte formation and swelling of the SM (7–9).

Due to the poor regenerative ability of AC and currently limited clinical treatments, there has been a growing interest in bioregenerative therapies, such as autologous conditioned serum, expanded or non-expanded mesenchymal stromal cells (MSCs) products and platelet concentrates (10). All of these therapies contribute to tissue regeneration, cartilage synthesis, inhibition of cartilage breakdown, and reduction in local inflammation (11, 12). Of these, IA infiltrations with platelet-rich plasma (PRP) and MSCs, more specifically adipose-derived mesenchymal stem cells (AMSCs), have emerged as treatments with encouraging results (13).

PRP contains different growth factors (GF) and proteins, such as fibronectin, vitronectin and fibrinogen, that play a significant role during AC repair and regeneration (14). Currently, IA infiltration is the most commonly administration route, which has shown to be effective in patients with mild OA; however, these therapies cannot reach the deeper layers of the AC and the SB, limiting their therapeutic potential (8, 15, 16). Furthermore, a recent study has shown the key role that SB plays in the pathogenesis of OA (17). Although literature is scarce, some authors have achieved positive outcomes after intra-osseous (IO) infiltration with PRP both in rabbits (18) and humans (9, 19–21).

MSCs have immunoregulatory and regenerative properties. They can stimulate the synthesis of angiogenic, mitogenic, and antiapoptotic mediators, encouraging the regeneration of damaged joint tissues (22). Additionally, MSCs can control the inflammation process by regulating the immune response (23). MSCs can be obtained from different tissues, including bone marrow, peripheral blood, umbilical cord, synovial fluid, placenta, and adipose tissue (AMSCs) (24). AMSCs have two major advantages: a greater proliferation capacity and an ability to maintain the differentiation potential for longer periods when cultured (25). Furthermore, adipose tissue is abundant and easy to obtain, and it has a lower risk of rejection as *in vitro* culture is not necessary (22, 26, 27). AMSCs secrete several anti-inflammatory substances, like interleukin-1 receptor antagonist, transforming growth factor β, stromal cell-derived factor 1 and others, which alleviate the inflammation state in the diseased joint (28). Different clinical trials on the use of AMSCs alone or in combination with PRP have demonstrated the safety and effectiveness of this therapy. No side effects have been reported, and a decrease in lameness and pain, along with an increase in joint functionality, were demonstrated. As a general trend, better outcomes were reported when the AMSCs were in combination with PRP (29–33). To our knowledge, no previous studies have described the IO injection of AMSCs to treat chondral defects.

On the other hand, several wet biomarkers have been identified and validated to facilitate the diagnosis of OA in the initial stages of the disease. These biomarkers can be detected in blood, urine, or synovial fluid and allow clinicians to identify patients at considerable risk for developing OA, control disease evolution and measure the response to treatments (34). Type II collagen cleavage neoepitope (C2C) is created by the cleavage of type II collagen by collagenases. It has been proven that C2C is increased in initial stages of OA, while it is decreased in chronic stages (35, 36). The utility of C2C concentration to monitor disease progression and response to treatment has been reported in several animal models, including dogs (37, 38), sheep (39), horses (40), mice (41), and guinea pigs (42). Another well-studied OA biomarker is hyaluronic acid (HA). Serum HA concentration is elevated in OA patients, and higher levels are associated with clinical and radiological worsening of pathology both in humans (43) and dogs (37, 44).

The present study hypothesizes that IA infiltration of platelet rich in growth factors (PRGF) together with IO infiltration of AMSCs, PRGF, or a combination of both could improve the therapeutic potential of IA-only PRGF infiltrations by targeting AC, SB, and SM. Based on this hypothesis, the aim of the study is (1) to assess the impact of IO infiltration of PRGF, AMSCs, or PRGF + AMSCs together with IA infiltration of PRGF in serum OA biomarkers (C2C and HA) in a model of acute full depth chondral defect in rabbits, and (2) to macroscopically assess the chondral defects using a validated scale (45). To our knowledge, this is the first study in which AMSCs and the combination of PRGF + AMSCs are IO infiltrated.

## Materials and methods

The study was approved by the Ethics Committee of Animal Welfare (CEEA) of the University CEU Cardenal Herrera of Valencia (Spain) in concordance to the Spanish Policy for Animal Protection (RD118/2021), which complies with European Union Directive 2010/63/UE with the following approval code: 2019/VSC/PEA/0153.

### Animals

A total of 80 healthy female New Zealand rabbits from the Polytechnic University in Valencia and specially bred for research purposes were used to accomplish a prospective randomized and blinded experimental study. The rabbits were 6 months old and with an average weight of 4.46 kg. The rabbits were allowed to eat, drink and move without restriction in individual big pens. All animals were monitored daily to detect signs of infection, pain and weight loss. To allow rabbits adaptation, an acclimatization period of 15 days prior to beginning the experiment was established. Inclusion criteria consisted of a normal physical examination and a normal hematology and serum biochemical analysis. All tests were performed 10 days before surgery, and results were within normal reference range values. No rabbits were excluded from the study.

During a 7-day period after the surgery, all animals received meloxicam 0.3 mg/kg SC q24h (Metacam^®^, Boehringer Ingelheim, Spain) and enrofloxacin 10 mg/kg SC q24h (Ganadexil^®^, Invesa, Spain). To evaluate the safety of the treatment, all animals were monitored daily, and a rabbit Grimace Scale was performed. If a score equal or >4 was obtained, buprenorphine 0.1 mg/kg SC q8h (Bupaq^®^, Richter Pharma AG, Austria) was given as a rescue analgesia. If during the study any animal showed drastic worsening in their physical or functional condition, they were excluded from the study. Severe and untreatable local infection and severe traumatic lesions (e.g., fractures) were considered exclusion criteria.

At the end of the study, all animals were euthanized in agreement with Spanish Policy for Animal Protection (RD118/2021). Rabbits were sedated with dexmedetomidine 0.05 mg/kg IM (Dexdomitor^®^, Esteve, Spain) and ketamine (10 mg/kg IM; Imalgene^®^, Merial, Spain), followed by an IV injection of pentobarbital sodium (150 mg/kg).

### Study groups

Rabbits were randomly divided into four groups of 20 animals, depending on the IO treatment administered:

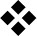
 Control group (CT): single IA injection of PRGF, followed by a single IO injection of saline solution.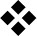 PRGF group: single IA injection of PRGF, followed by a single IO injection of PRGF.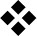 AMSCs group: single IA injection of PRGF, followed by a single IO injection of AMSCs.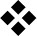 PRGF + AMSCs group: single IA injection of PRGF, followed by a single, combined IO injection of PRGF and AMSCs.

Additionally, animals were divided into two subgroups of ten animals each, according to the survival time (56 or 84 days).

### Plasma rich in growth factors and adipose derived mesenchymal stromal cells

To obtain an autologous preparation of PRP, PRGF-Endoret^®^ technology was used. After sedating the animals with IM dexmedetomidine (0.05 mg/kg; Dexdomitor^®^, Esteve, Spain), IM ketamine (10 mg/kg; Imalgene^®^, Merial, Spain), and IM morphine (1 mg/kg; B-Braun^®^, Germany), a total of 15 mL of blood were collected from the auricular artery of each rabbit under sterile conditions in vacutainer sodium citrate 3.8% tubes (BD Vacutainer^®^ 9NC, New Jersey, USA). The tubes were centrifuged at 460 g for 8 min (PRGF^®^ System III, Biotechnology Institute^®^, Álava, Spain) to separate the PRGF from the platelets poor plasma, the white blood cells and the red blood cells. A sterile pipette was used to collect the PRGF fraction and transfer it into a sterile tube, where platelets were activated for GF release just prior to infiltration by adding 10% calcium chloride (50 μL/mL of PRGF).

### Adipose derived mesenchymal stromal cells

Allogenic AMSCs from an individual donor rabbit from Principe Felipe Research Centre were used. A total of 30 g of inguinal adipose tissue were obtained. Moreover, a 20 mL blood sample was collected into blood collection tubes without anticoagulant.

Adipose tissue was washed with Phosphate Buffered Saline Solution (PBS) and incubated in a PBS solution containing penicillin, streptomycin and type 1-A collagenase and dispase. The tissue was then digested overnight at 37°C. The next day, the adipose tissue was washed, and the obtained stromal vascular fraction was cultured in an autologous serum. The cells were allowed to grow to 1 × 10^6^ cells per gram and were then amplified during four passages. Thereafter, cells were resuspended in 10% PBS supplemented DMEM for tissue transplantation.

Cell viability was assessed by using trypan blue dye, and cell viability was >90%.

### Chondral defect model and treatments

After sedation and blood extraction, the medial aspect of both hindlimbs was prepared for aseptic surgery. General anesthesia was mask induced and maintained with sevoflurane (Sevoflo^®^, Esteve, Spain).

**Figure 1:**
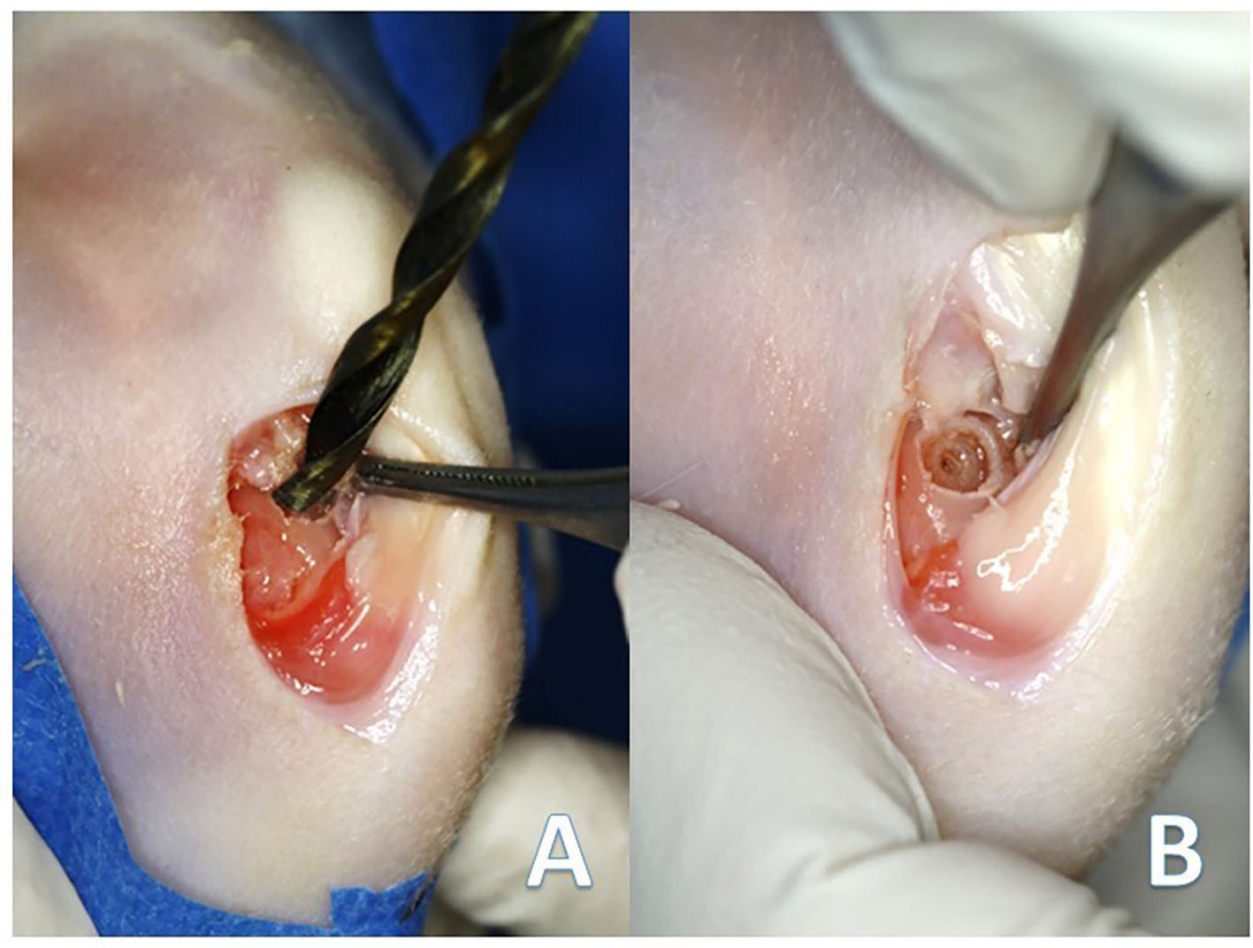
**(A)** Creation of the chondral defect in the medial femoral condyle of the rabbits with the drill bit. **(B)** Final defect 4 mm in diameter and 5 mm in depth.

With the rabbit's knee in complete flexion, a skin incision of 10 mm long was performed over the margin of the medial femoral condyle, followed by an incision of the fascia and joint capsule to expose the medial femoral condyle. The loading area was then identified, and a defect of 4 mm in diameter and 5 mm in depth was created with a drill bit (Figure 1). Finally, simple stitches were used to close the different layers using 3/0 polyglyconate (Novosyn^®^ Quick, B-Braun, Germany). The same procedure was performed in the contralateral knee.

**Figure 2:**
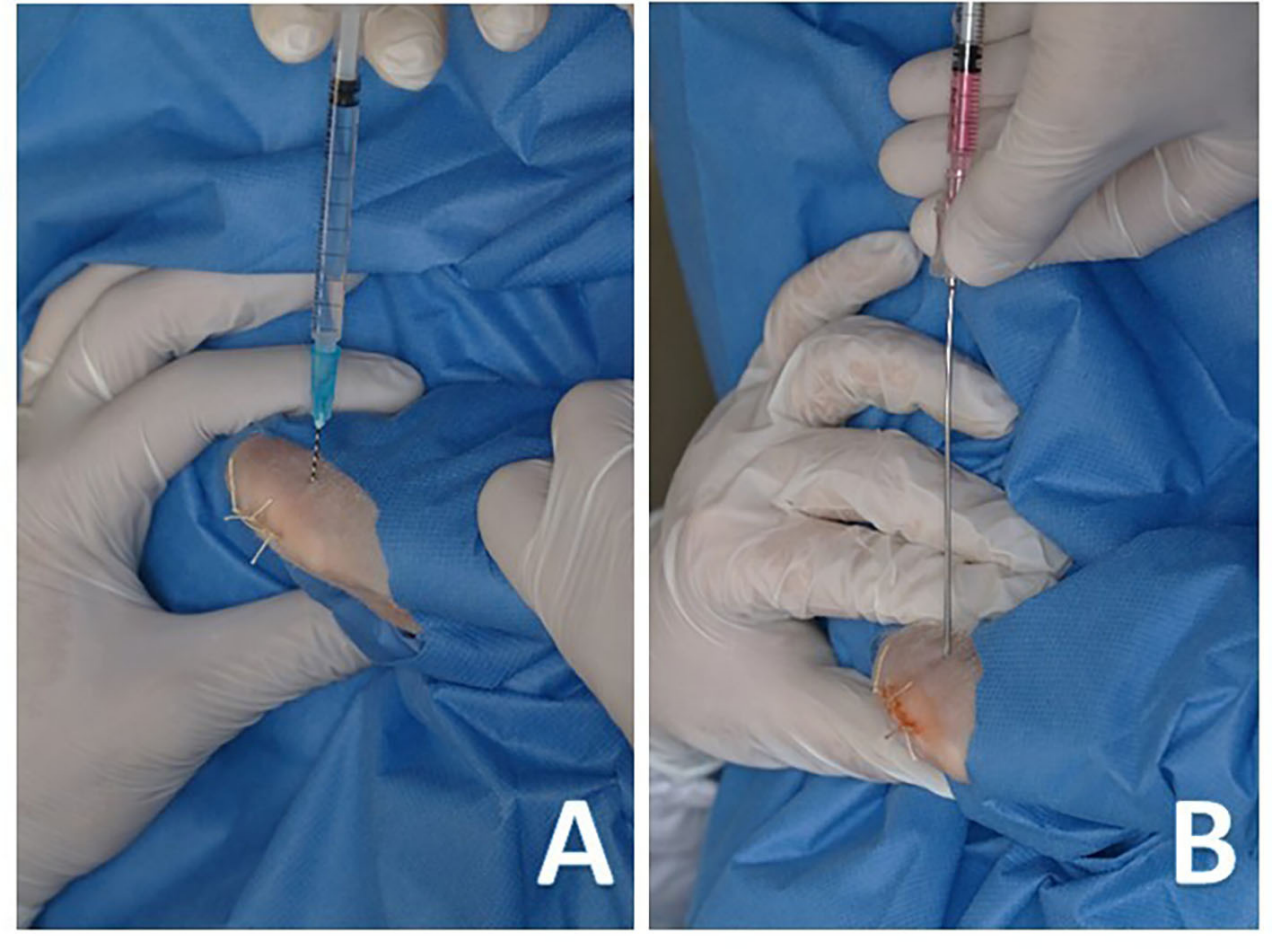
**(A)** Intra-articular infiltration of plasma rich growth factors with a 22-G hypodermic needle. **(B)** Intra-osseous infiltration of adipose derived mesenchymal stem cells with an 18-G spinal needle.

After surgery, IA infiltration of both knees with PRGF was performed using a 22-G needle. The needle was inserted laterally to the patellar tendon, while the knee was kept in flexion, and a single infiltration with 0.25 mL of PRGF was performed in all animals (Figure 2A) regardless of the treatment group. Finally, the IO infiltration occurred by inserting an 18-G spinal needle perpendicular to the femur in the lateral supracondylar area with gentle rotating movements. This was followed by a single infiltration of 0.5 mL saline solution, PRGF, AMSCs, or PRGF + AMSCs, depending on the treatment group (Figure 2B). A mean time of 29.16 min elapsed from blood collection to PRGF injection.

### Serum biomarkers study

A total 3.5 mL of blood was collected from the auricular artery of each rabbit in vacutainer serum tubes with a clot accelerator and a gel serum separator (BD Vacutainer^®^ SST™ II Advance, BD, New Jersey, USA). Blood samples were extracted just prior to surgery (baseline) and 28-, 56-, and 84-days after infiltration.

After centrifugation of the blood samples at 3,000 g for 5 min, the obtained serum was frozen in Eppendorf tubes at −80°C. To determine the serum concentration of C2C and HA, enzyme-linked immunosorbent assay (ELISA) tests IBEX^®^ 60-1001-001 and TECO^®^ TE1017-2 were used, respectively.

### Macroscopical assessment

After the animals were euthanized (56- and 84- days after infiltration), both right and left femurs were excised to perform the macroscopical assessment. Cartilage repair was graded by four blinded observers with experience in cartilage research. Goebel et al. semiquantitative scale was followed. The scale consists of five major parameters and 25 items, where 20 points is the worst possible result (45).

### Statistical analysis

The sample size was calculated by a power analysis consistent with results published in prior research in which only IA PRP was administered (mean = 10.83 and standard deviation = 2.041) (46). An alpha level of 0.02 and a power of 80% were established.

The data were processed using the SPSS 20.0 program for Windows (SPSS^®^Inc., Chicago, USA). For each variable, a descriptive study of the mean, standard deviation and confidence intervals was performed. A *p*-value < 0.05 was considered significant. The Shapiro-Wilk test was used to assess the normality of data in every quantitative variable, while the Leven test was used to assess the variance homogeneity. All variables adhered to a normal distribution; hence, a mixed ANOVA test was used to compare the variables.

## 3. Results

### 3.1. Treatment safety

A total of 78 rabbits completed the study (97.5% of the animals). Two rabbits were excluded from the study due to a severe worsening of their physical condition. One of them was excluded due to a severe infection in the surgical area with multidrug resistant *Pseudomonas aeruginosa*. The other animal had a molar abscessation, which caused extreme weight loss.

### 3.2. Serum biomarkers assessment

#### 3.2.1. Type II collagen neoepitope

**Figure 3:**
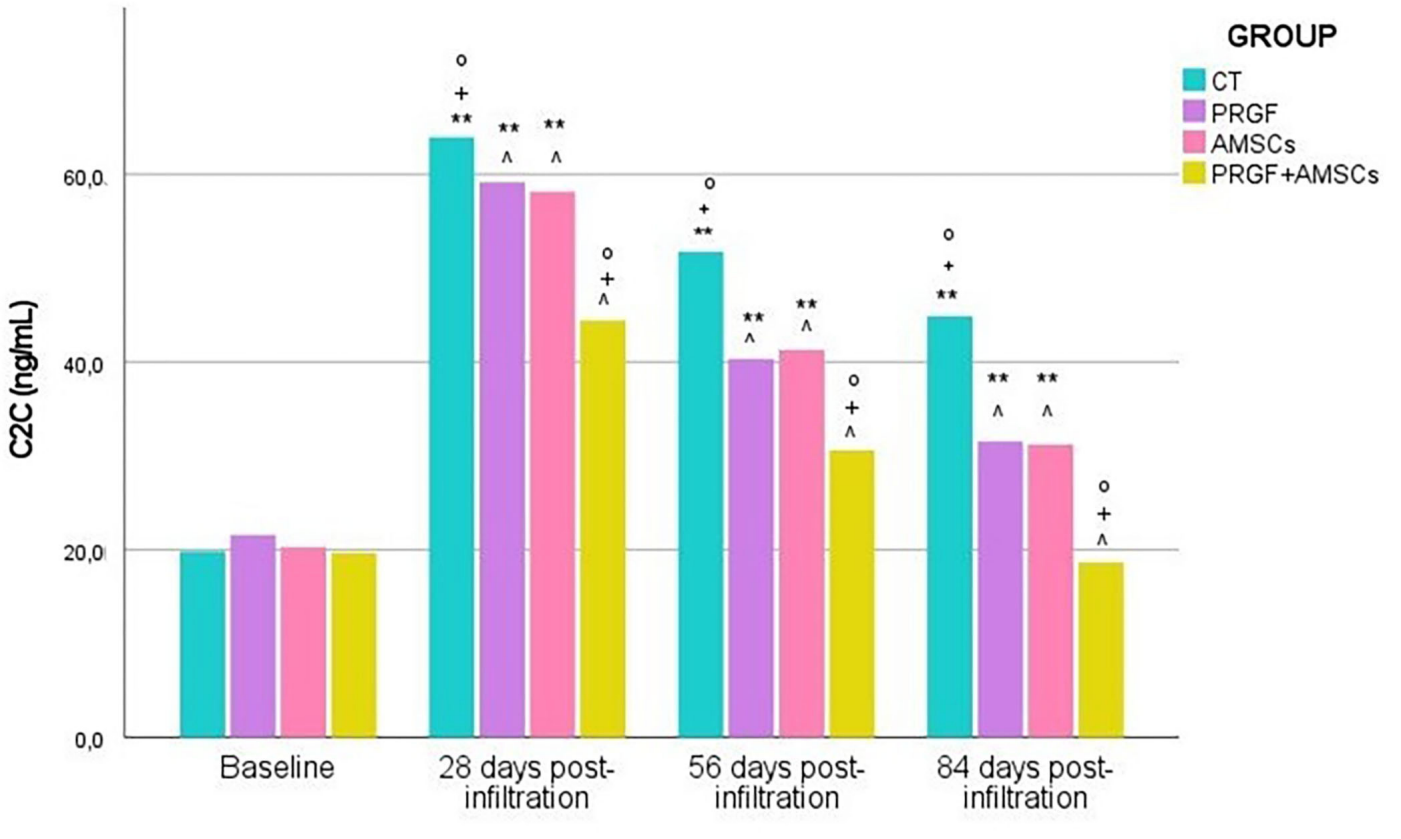
C2C serum concentration (ng/mL) before infiltration and 28-, 56-, and 84-days post-infiltration in all treatment groups. C2C, type II collagen cleavage neoepitope; CT, control; PRGF, plasma rich in growth factors; AMSCs, adipose mesenchymal stem cells; PRGF + AMSCs, combination of plasma rich growth factors with adipose mesenchymal stem cells. ∧, significant differences with CT group; °, significant differences with PRGF group; +, significant differences with AMSCs group; **, significant differences with PRGF + AMSCs group.

No significant differences (*p*-value = 0.509) in C2C serum concentration between groups were demonstrated at baseline. However, significanty higher values were observed 28 days after infiltration in the CT group than in the PRGF (*p*-value =0.009), AMSCs (*p*-value = 0.007), and PRGF + AMSCs (*p*-value < 0.001) groups. No significant differences between PRGF and AMSC groups (*p*-value = 0.925) were demonstrated, and the PRGF + AMSCs group showed significantly lower C2C values than the PRGF and AMSCs groups (*p*-value < 0.001) (Figure 3).

Similar results were shown 56 days after infiltration. CT group showed significantly higher values (*p*-value < 0.001) of C2C than the PRGF, AMSCs, and PRGF + AMSCs groups. The group treated with PRGF + AMSCs showed significantly lower concentrations of C2C (*p*-value < 0.001) than the PRGF and AMSCs groups; nevertheless, no significant differences were reported between the PRGF and AMSCs groups (*p*-value = 0.936) (Figure 3).

At 84 days follow-up, the CT group showed significantly higher C2C concentrations than the PRGF, AMSCs, and PRGF + AMSCs groups (*p*-value < 0.001). Moreover, the PRGF + AMSCs group showed significantly lower concentrations than the PRGF and AMSCs groups (*p*-value < 0.001), while no differences were seen between the PRGF and AMSCs groups (*p*-value = 0.997) (Figure 3).

A significantly higher C2C concentration level was reported in the CT (*p*-value < 0.001), PRGF (*p*-value = 0.032), and AMSCs (*p*-value = 0.028) groups 84 days after infiltration than at baseline. However, no significant differences were observed in the PRGF + AMSCs group between baseline and 84 days after infiltration (*p*-value = 0.428).

The mean and standard deviation of C2C serum concentration (ng/mL) in different treatment groups at each study time are described in Table 1.

**Table 1:** Serum C2C means standard deviation at different study times and treatment groups.

**Study time**	**CT**	**PRGF**	**AMSCs**	**PRGF + AMSCs**
Baseline	19.80 ± 2.46	21.59 ± 3.70	20.27 ± 4.70	19.66 ± 2.54
28 days post-infiltration	63.94 ± 3.92	59.16 ± 3.65	58.15 ± 3.31	44.43 ± 2.98
56 days post-infiltration	51.75 ± 3.44	40.34 ± 3.36	41.29 ± 3.50	30.61 ± 2.94
84 days post-infiltration	44.89 ± 2.94	31.54 ± 2.84	31.20 ± 4.02	18.66 ± 3.17

CT, control; PRGF, plasma rich in growth factors; AMSCs, adipose mesenchymal stromal cells; PRGF + AMSCs, combination of plasma rich growth factors with adipose mesenchymal stromal cells.

#### 3.2.2. Hyaluronic acid

**Figure 4:**
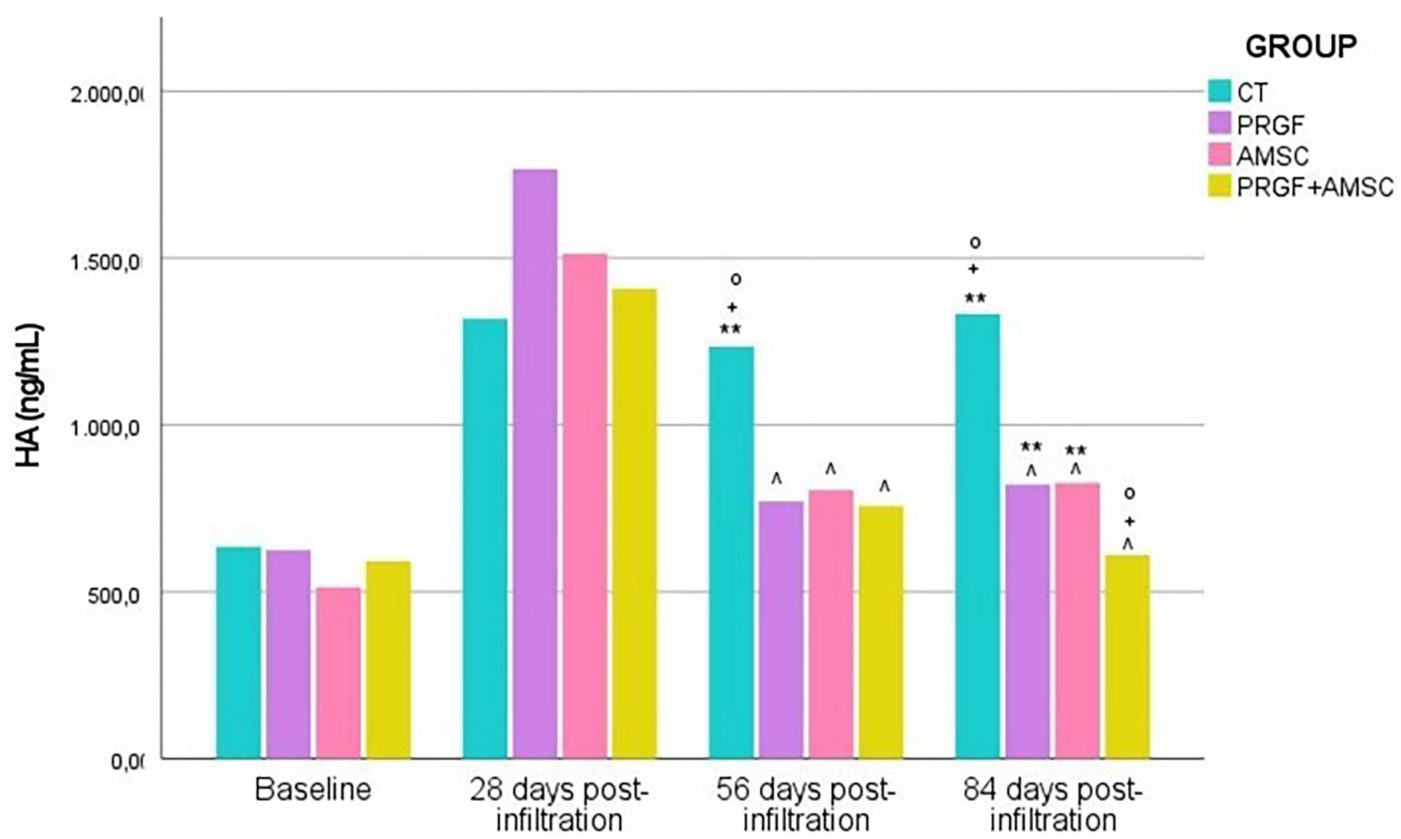
HA serum levels (ng/mL) before infiltration and 28-, 56-, and 84-days post-infiltration in all treatment groups. HA, hyaluronic acid; CT, control; PRGF, plasma rich growth factors; AMSCs, adipose mesenchymal stem cells; PRGF + AMSCs, combination of plasma rich in growth factors with adipose mesenchymal stem cells. ∧, significant differences with CT group; °, significant differences with PRGF group; +, significant differences with AMSCs group; **, significant differences with PRGF + AMSCs group.

No significant differences in HA concentrations were demonstrated between groups at baseline (*p*-value= 0.091) nor 28 days after the infiltration (*p*-value = 0.460), except higher concentrations were reported in the PRGF group. However, 56 days after infiltration, the CT group showed significantly higher values of HA concentrations (*p*-value < 0.001) than the PRGF, AMSCs, and PRGF + AMSCs groups (Figure 4).

On the other hand, 84 days after the infiltration significantly higher HA concentration were observed in the CT group (*p*-value < 0.001). No significant differences between the PRGF and AMSCs groups were reported (*p*-value = 1.00); nevertheless, the PRGF + AMSCs group showed significantly lower values than the PRGF (*p*-value = 0.010) and AMSCs (*p*-value = 0.006) groups (Figure 4).

No significant differences between baseline HA concentration values and 84 days after treatment values were reported in the PRGF + AMSCs treatment group (*p*-value = 0.214). However, significantly higher values have been reported in the CT (*p*-value < 0.01), PRGF (*p*-value= 0.042), and AMSCs (*p*-value = 0.023) groups at the end of the study compared to baseline values.

The mean and standard deviation of serum HA concentrations (ng/mL) in different treatment groups at each study time are described in Table 2.

**Table 2:** Serum HA means standard deviation at different study times and treatment groups.

**Study time**	**CT**	**PRGF**	**AMSCs**	**PRGF + AMSCs**
Baseline	635.67 ± 76.64	625.18 ± 114.26	513.87 ± 174.65	592.13 ± 86.57
28 days post-infiltration	1,319.04 ± 524.36	1,767.22 ± 936.37	1,513.78 ± 716.37	1,408.60 ± 533.21
56 days post-infiltration	1,234.96 ± 391.67	770.99 ± 104.67	804.99 ± 50.21	757.93 ± 46.12
84 days post-infiltration	1,333.54 ± 265.19	821.90 ± 43.29	826.92 ± 59.66	610.05 ± 64.44

CT, control; PRGF, plasma rich in growth factors; AMSCs, adipose mesenchymal stromal cells; PRGF + AMSCs, combination of plasma rich in growth factors with adipose mesenchymal stromal cells.

### 3.3. Macroscopical assessment

#### 3.3.1. Fifty-six days after infiltration

**Figure 5:**
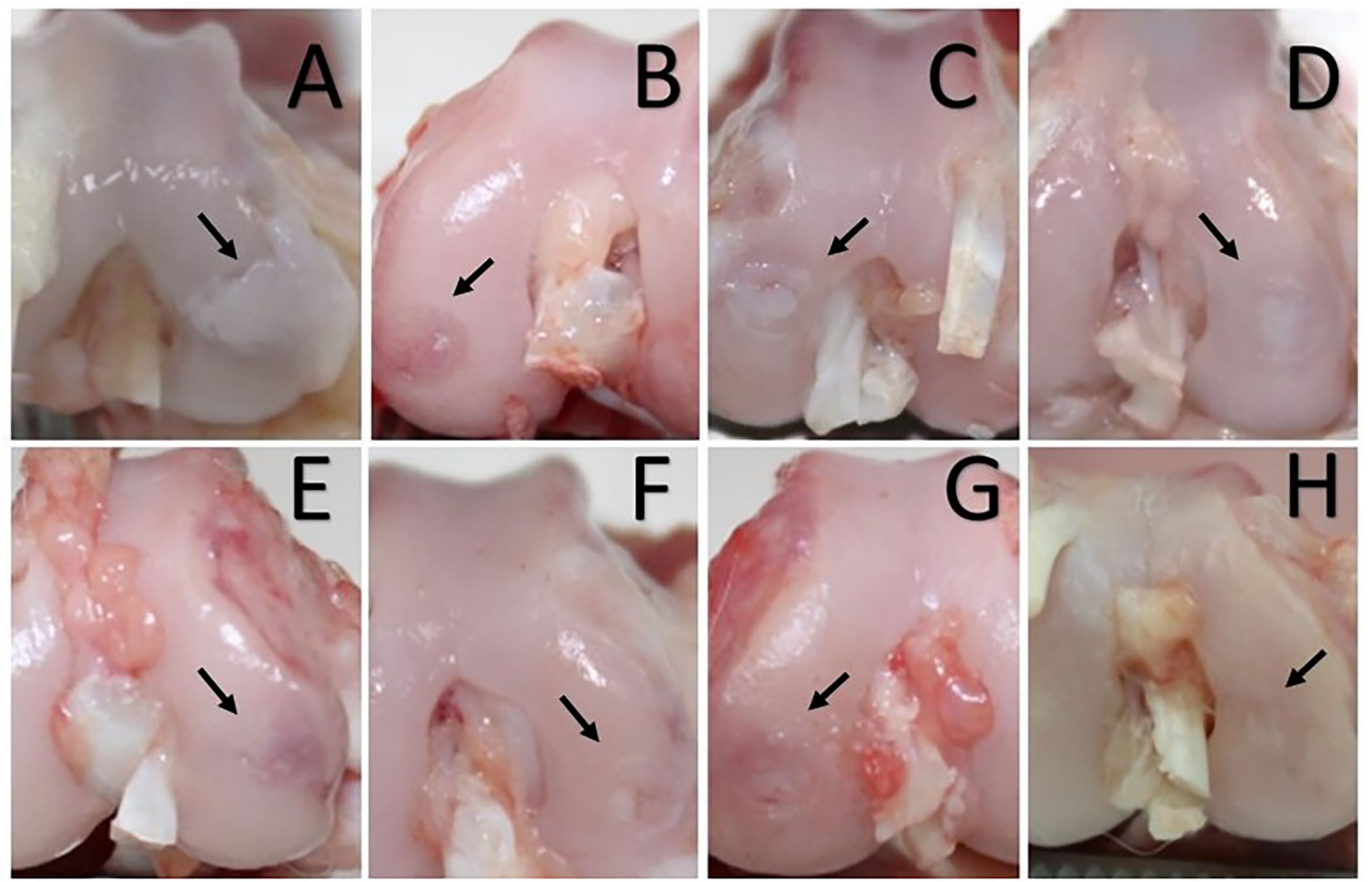
Chondral defect assessment 56 **(A–D)** and 84 days **(E–H)** after the intraosseous infiltration. **(A, E)** Control group. **(B, F)** Platelet rich in growth factors group treated condyles. **(C, G)** Adipose mesenchymal stem cells treated condyles. **(D, H)** Condyles treated with the combination of plasma rich in growth factors and adipose mesenchymal stem cells. **(A)** White and hypertrophic repair tissue with cracks and fibrillations in the adjacent cartilage. **(E)** Translucent, and fibrillated tissue with diffuse osteoarthritic changes in the adjacent articular cartilage. **(B, C)** Predominantly translucent and smooth repair tissue. More than 50% of the defect's depth filled in, and blood vessels are present in more than 50% of the repaired tissue. **(F, G)** Predominantly white and smooth repaired tissue; the defect is almost level with the adjacent cartilage. Some cracks are present in the adjacent articular cartilage. **(D)** Predominantly white, smooth, and homogeneous repair tissue; the defect is level with the adjacent articular cartilage, which looks normal. **(H)** Hyaline, smooth, and homogeneous repair tissue. The defect is filled and level with adjacent articular cartilage.

**Figure 6:**
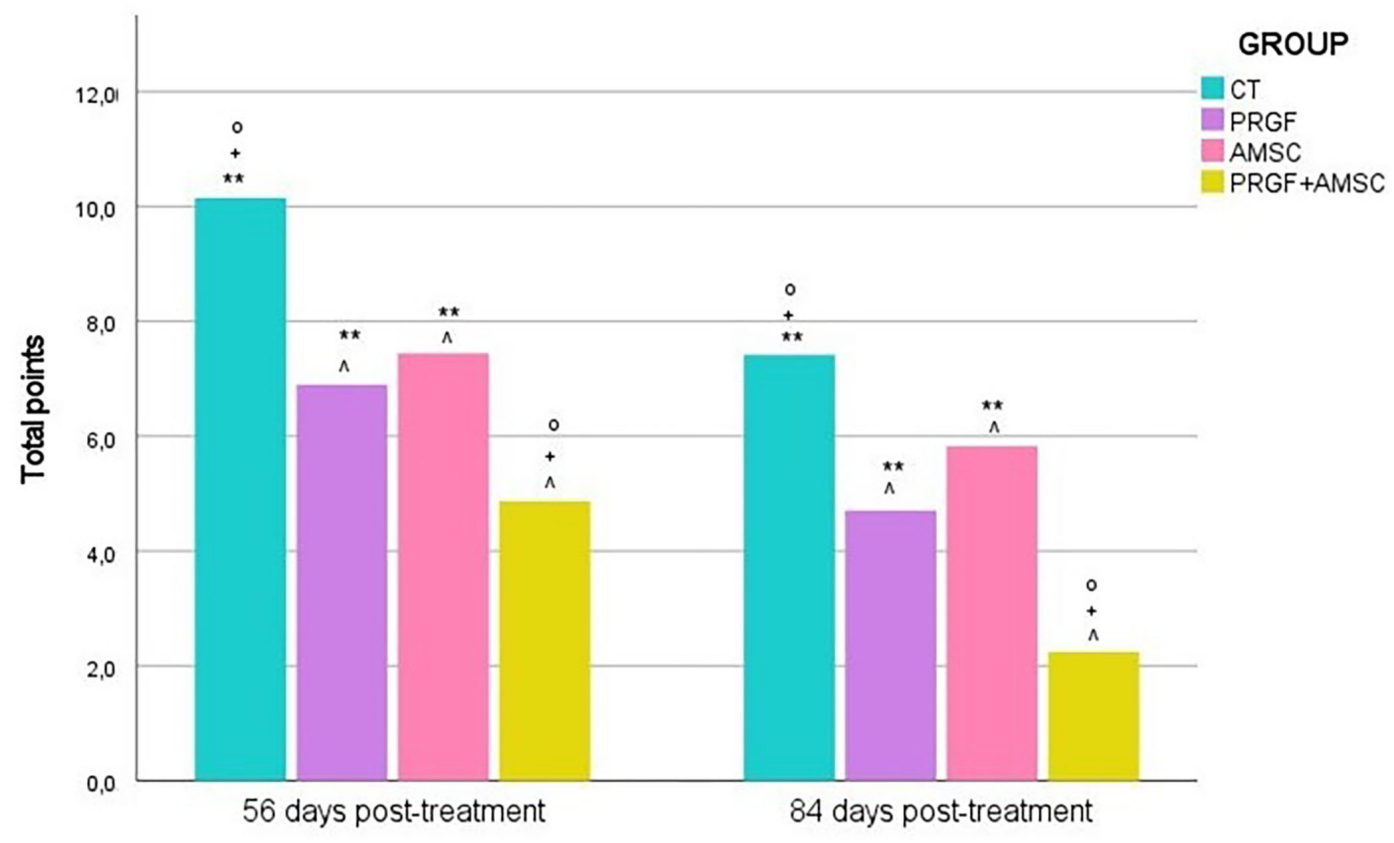
Macroscopic assessment of the chondral defect 56 and 84 days after the intraosseous infiltration following the Goebel et al. scale. Total points received by each treatment group. Significantly higher scores are shown by CT group, followed by the AMSC and PRGF groups, without significant differences between them. PRGF + AMSCs group achieved a significantly lower score. ∧, significant differences with CT group; °, significant differences with PRGF group; +, significant differences with AMSCs group; **, significant differences with PRGF + AMSCs group.

Cartilage treated with the combination of PRGF + AMSCs showed a significantly lower macroscopical assessment total score compared to the CT (*p*-value < 0.001), PRGF (*p*-value = 0.025), and AMSCs (*p*-value = 0.004) groups (Figures 5, 6). Additionally, no significant differences were observed between the PRGF and AMSCs groups (*p*-value = 0.641), but both groups showed significantly lower total scores compared to the CT group (*p*-value = 0.001 and 0.011, respectively).

Means and standard deviations for each evaluated parameter and total points are shown in Table 3.

**Table 3:** Macroscopical scores 56 days after infiltration.

**Parameter**	**CT**	**PRGF**	**AMSCs**	**PRGF + AMSCs**
Color of the repair tissue	2.11 ± 0.69	1.21 ± 0.48	1.32 ± 0.30	0.62 ± 0.21
Presence of blood vessels in the repair tissue	0.92 ± 0.99	2.33 ± 0.53	1.25 ± 0.46	1.62 ± 0.52
Surface of the repair tissue	2.31 ± 1.07	1.08 ± 0.75	1.62 ± 0.28	0.79 ± 0.35
Filling of the defect	2.27 ± 0.66	1.12 ± 0.50	1.83 ± 0.25	0.83 ± 0.19
Degeneration of adjacent articular cartilage	1.95 ± 0.65	1.21 ± 0.30	1.41 ± 0.23	0.99 ± 0.44
Total points	9.57 ± 1.75	6.96 ± 1.41	7.44 ± 0.71	4.87 ± 0.47

CT, control; PRGF, plasma rich in growth factors; AMSCs, adipose mesenchymal stromal cells; PRGF + AMSCs, combination of plasma rich in growth factors with adipose mesenchymal stromal cells.

Mean and standard deviation.

#### 3.3.2. Eighty-four days after infiltration

Cartilage treated with the combination of PRGF + AMSCs showed a significantly lower macroscopical assessment total score compared to the CT (*p*-value < 0.001), PRGF (*p*-value < 0.001), and AMSCs (*p*-value < 0.001) groups (Figures 5, 6). Additionally, no significant differences were observed between the PRGF and AMSCs groups (*p*-value = 0.198). Moreover, the PRGF and AMSCs groups showed significantly lower total scores compared to the CT group (p-value < 0.001 and = 0.032, respectively).

Means and standard deviations for each evaluated parameter and total points are shown in Table 4.

**Table 4:** Macroscopical scores 84 days after infiltration.

**Parameter**	**CT**	**PRGF**	**AMSCs**	**PRGF + AMSCs**
Color of the repair tissue	1.55 ± 0.57	0.87 ± 0.52	0.98 ± 0.44	0.44 ± 0.27
Presence of blood vessels in the repair tissue	0.79 ± 0.40	0.89 ± 0.45	0.58 ± 0.35	0.23 ± 0.17
Surface of the repair tissue	1.78 ± 0.41	0.89 ± 0.57	1.47 ± 0.39	0.50 ± 0.28
Filling of the defect	1.57 ± 0.39	0.98 ± 0.43	1.53 ± 0.36	0.51 ± 0.34
Degeneration of adjacent articular cartilage	1.61 ± 0.35	1.09 ± 0.49	1.23 ± 0.22	0.57 ± 0.47
Total points	7.26 ± 1.02	4.71 ± 1.66	5.79 ± 0.82	2.24 ± 0.99

CT, control; PRGF, plasma rich in growth factors; AMSCs, adipose mesenchymal stromal cells; PRGF + AMSCs, combination of plasma rich in growth factors with adipose mesenchymal stromal cells.

Mean and standard deviation.

## Discussion

During the last decade, bioregenerative therapies, such as MSCs and PRP, have been used to treat several musculoskeletal conditions, including chondral defects and OA. PRP and MSCs are commonly IA infiltrated. Even though promising results have been reported in patients with mild OA, in severely affected patients therapeutic potential is limited, as these regenerative therapies cannot reach the deeper layers of AC or the SB (47). Thus, it has been postulated that IA infiltrations combined with IO infiltrations could target SB, providing a more comprehensive treatment (48, 49).

IO injections with bone marrow concentrate have been proposed to treat knee OA in humans and some success in pain relief, functionality and lack of subsequent surgery has been achieved (48, 50). However, to our knowledge, no article on IO infiltration of AMSCs has been published either in animals or in humans.

The literature regarding IO injections of PRP to treat severe OA is also sparse, and to our knowledge, no treatment related to veterinary medicine has been published. The IO infiltration technique was introduced by Sánchez et al. in 2014 (9), and since then, some pilot studies in humans have reported positive outcomes, such as pain relief and joint function improvements (9, 19, 20, 47, 51, 52). These positive clinical outcomes could be correlated with the significantly better macroscopical regeneration of the AC shown in our study in PRGF, AMSCs, and PRGF + AMSCs groups. Nonetheless, further studies to compare the macroscopical assessment with the histology of the regenerated tissue are needed.

A total lower macroscopical score was achieved in the PRGF + AMSCs group, followed by the PRGF, AMSCs, and CT groups. Regarding the color of the repair tissue, 56- and 84-days after infiltration the PRGF + AMSCs group showed a hyaline color, while the PRGF and AMSCs groups showed predominantly white color with some translucent areas. On the other hand, the CT group showed mainly translucent color. Fifty-six days after treatment blood vessels were present in more than 50% of the repair tissue in the PRGF and PRGF + AMSCs groups. Our research team believes that the lower total scores shown in the PRGF + AMSCs and PRGF treatment groups, meaning a faster and improved regeneration process, are correlated with the increased presence of blood vessels showed in the macroscopical scales in these groups. The angiogenic activities of PRP are modulated by stimulatory pro-angiogenic platelet-derived vascular endothelial growth factor, together with several other mediators in PRP, such as fibroblast growth factor-2 and transforming growth factor-β, that stimulate endothelial cells to produce new blood vessels (53). This angiogenesis process is known to be essential for tissue healing to withstand the high metabolic activity of the AC repair process (54). Regarding the surface of the repair tissue, predominantly fibrillated tissue was present in the CT group at both study times, while in the PRGF + AMSCs group a smooth and homogeneous tissue was present. Moreover, <50% of the depth of the defect was filled in the CT group at 56 days follow-up, in contrast to the PRGF, AMSCs and PRGF + AMSCs groups, where more than 50% of the lesion was filled. At 84 days follow-up, the defect in the PRGF + AMSCs group was level with the adjacent cartilage. The adjacent cartilage in the CT group showed diffuse osteoarthritic changes at both study times, while the PRGF and AMSCs groups showed cracks and fibrillations in the integration zone and the PRGF + AMSCs group showed an almost normal appearance.

On the other hand, Muinos-Lopez et al. assessed the effect of IA or a combination of IA and IO infiltrations of PRP on the cellular content of synovial fluid (55). Synovial fluid was collected before and 1 week after the infiltration and the presence of MSCs, monocytes and lymphocytes were determined. It was concluded that the IO infiltration induced a greater reduction in the population of monocytes and lymphocytes, and an increase in MSCs modulation was observed when the PRP was directly infiltrated in the SB (55). Lychagin et al. additionally assessed the inflammatory response by quantifying an OA biomarker (serum oligomeric matrix protein) level and concluded that the concentration was initially higher in osteoarthritic patients than in healthy control patients. These levels continue to increase 1 and 3 months after the IO infiltration, but posterior analysis showed constant serum oligomeric matrix protein levels (51).

Similar results have been obtained in our study, where serum C2C and HA concentrations were assessed. In the present study, an increase in serum C2C concentration was reported 28 days after the creation of the chondral defect in all study groups, matching the acute damage. Moreover, at 56 days follow-up, serum C2C concentration started to decrease in all study groups, and this decrease still was evident at 84 days follow-up. Significantly higher serum levels were observed in the CT group at all study times, while the PRGF + AMSCs group showed the lowest levels, followed by the PRGF and AMSCs groups without significant differences between the last two. No significant differences were reported between baseline and 84 days follow-up values in the PRGF + AMSCs group; however, in the other three treatment groups, serum levels 84 days after the infiltration remained statistically higher than baseline levels. These results suggest that IA PRGF combined with IO PRGF + AMSCs infiltration improve cartilage healing more than IA infiltration of PRGF or AMSCs itself. Furthermore, the IO combination of PRGF and AMSCs have shown better results than the IO infiltration of one of these therapies by itself. Comparable results were observed in our previous study, in which lower C2C serum concentration was reported in treatment groups 84 days after the infiltration, compared to the CT group. Moreover, the PRGF group showed stable concentrations at 84 days follow-up, while in the CT group C2C levels continued to rise (18).

On the other hand, statistically lower serum HA levels have been reported in the CT group at 28 days follow-up, while the PRGF group showed the highest concentration, followed by the AMSCs and the PRGF + AMSCs groups. However, 56 and 84 days after the infiltration, the CT group showed statistically higher values than the other groups. A sudden fall of serum HA levels in the PRGF, AMSCs, and PRGF + AMSCs groups was reported at 56 days follow-up, as it did in our previous study (18). This great rise, followed by the sudden fall of HA levels in animals treated with IO PRGF or AMSCs might be suggestive of a faster chondrogenesis process. Additionally, at 84 days follow-up, the PRGF + AMSCs group showed statistically lower HA concentrations than the CT, PRGF, and AMSCs groups, and no significant differences between baseline values and 84 days follow-up values were reported in this group.

In the present study, significantly better results have been reported in the PRGF + AMSCs group, in concordance with previous studies in which this combination has shown some beneficial effects, such as enhanced proliferation of MSCs and improvements in joint function (29, 30, 56). Additionally, this combination has shown enhanced collagen type II expression and reduced chondrocyte apoptosis. Moreover, PRP has an anabolic effect on both MSCs and chondrocytes, prompting proliferation and strengthening matrix development, together with an increase in proteoglycans concentration in AC (57–59).

Although several studies have shown that IO approach is safe, the technique has some limitations. It is an invasive procedure, and it should be performed in the operating room with the aid of ultrasound or fluoroscopy. Sedation and general or local anesthesia are required, making the technique more expensive, and animal owners might be reluctant to consent to the treatment.

This study is one of the first experimental animal studies in this subject; moreover, it is one of the first in which objective records have been assessed. The major limitation of the study is the short follow-up period, and the paucity of experimental studies with which to compare our results. Despite the positive results that IO infiltration with PRGF, AMSCs or a combination of both have shown, further studies are required to evaluate histological and biomechanical alterations. Additionally, further clinical trials in companion animals are needed, opening the door to further research in this field.

## Conclusions

The combination of IA infiltrations of PRGF with IO infiltration of PRGF, AMSCs or a mixture of both targets the AC, the SM and the SB tissues implicated in the development of OA. Thus, the IO administration method is a potential tool in managing full depth chondral defects or severe OA.

Better outcomes have been observed in rabbits treated with IA PRGF together with IO PRGF, AMSCs or a combination of PRGF + AMSCs, and no clinical complications have been reported. Nevertheless, further studies in this field are necessary to link these results with histological, biomechanical, and clinical outcomes. No adverse effects were reported during the study time, suggesting IO infiltration is a safe treatment strategy.

## Data availability statement

The raw data supporting the conclusions of this article will be made available by the authors, without undue reservation.

## Ethics statement

The study was conducted according to the guidelines of the Declaration of Helsinki and approved by the Ethics Committee of Animal Welfare (CEEA) of the University CEU Cardenal Herrera of Valencia (approval code: 2019/VSC/PEA/0153) and was conducted in accordance with the ARRIVE (Animal Research: Reporting *in vivo* Experiments) guidelines.

## Author contributions

JS and JCa: conceptualization. JS and MR: methodology and visualization. MR: software. MR, JS, ED, and JCa: validation. JCe and MT-T: formal analysis. MT-T, LM-P, BC, ED, and PP: investigation. JS: resources and project administration. MT-T, PP, and JCa: data curation. MT-T: writing—original draft preparation. MT-T and ED: writing—review and editing. ED and MR: supervision. JCa: funding acquisition. All authors have read and agreed to the published version of the manuscript.
